# Retrospective evaluation of Penguin Cold Caps for chemotherapy-induced alopecia

**DOI:** 10.1007/s00520-024-08393-7

**Published:** 2024-03-13

**Authors:** Dale Weaver, Michelle L. Pershing, Bethany Golden, Laura Hammel, Pauline Kefalas Russ, Mark Cripe

**Affiliations:** 1 OhioHealth Cancer Care, 3535 Olentangy River Road Columbus, Columbus, OH 43214 USA; 2 OhioHealth Research Institute, 3545 Olentangy River Road Columbus, Columbus, OH 43214 USA; 3 Over My Head, 500 Thomas Lane Columbus, Columbus, OH 43214 USA; 4 OhioHealth Cancer Care, 3430 OhioHealth Parkway Columbus, Columbus, OH 43202 USA; 5 OhioHealth Breast Surgeons, 285 East State Street, Suite 300, Columbus, OH 43215 USA

**Keywords:** Chemotherapy, Chemotherapy-induced alopecia, Penguin Cold Caps

## Abstract

**Background:**

Scalp cooling is an increasingly recognized non-pharmacologic approach to minimize chemotherapy-induced alopecia (CIA). Several commercially available machine-based and manual scalp cooling systems are available; however, literature reports of effectiveness are highly variable. The purpose of this study was to determine real-world tolerability and subjective effectiveness of a manual cold capping system in minimizing CIA across a variety of patient race and hair types. This study was a single-institution review of outcomes from manual cold capping.

**Methods:**

We identified retrospective cohort of adult patients who presented to discuss cold capping between January 14, 2019, and March 31, 2022. Data collected from medical records included demographics, decision to pursue/continue cold capping, diagnoses, chemotherapy regimens, hair characteristics (length, thickness, coarseness, type), and subjective perception of percentage of hair retained. Those with successful vs. unsuccessful cold capping (≥ 50% vs. < 50% of hair retained) were compared based on the patient-level factors of interest.

**Findings:**

A total of 100 patients initiated cold capping during the study period, and 95% of them completed cold capping. The majority of patients who started cold capping completed it. The median-reported percentage of hair maintained was 75%, and 82/89 (92.1% of patients) had favorable results, defined as ≥ 50% of hair retained. The only patient-level factor associated with favorable response was chemotherapy regimen, with fewer patients receiving doxorubicin-containing regimens having successful hair retention compared to other chemotherapy types (71.4% successful results vs. 95.7% for those receiving paclitaxel-containing regimens and 96.6% for those receiving docetaxel-containing regimens (*p* = 0.018). There was no difference in success based on patient race/ethnicity or hair characteristics.

**Interpretation:**

The overall effectiveness (92.1%) in this study is consistent to higher than many literature reports. One possible reason for the high success in our cohort is compliance with cold capping protocols, meaning applying the cap in the appropriate manner and wearing the cap for the prescribed durations, which may impact effectiveness.

## Introduction

This study was a single-institution review of outcomes from manual cold capping. The majority (65–80% or more) of those who undergo chemotherapy can expect some degree of chemotherapy-induced alopecia (CIA) [1–3]. Chemotherapy attacks rapidly dividing cells in the body, including the hair follicles [4]. CIA is often ranked among the most distressing side effects, with potentially significant impact on quality of life and overall wellness during the cancer journey [5, 6]. Some patients have refused chemotherapy because of the CIA [7, 8]. Many methods to save hair have been tested, but scalp cooling has been the most effective in reducing CIA [7]. Scalp cooling is a non-medicinal approach to minimize CIA, whereby ice packs or cooling caps are placed on the scalp for up to an hour before, during, and up to 7 h after each chemotherapy session, depending on the chemotherapy regimen and cooling system [9]. The cold results in local vasoconstriction, which potentially minimizes the amount of cytotoxic drug that reaches the cells of the hair follicles [9, 10].

Scalp cooling has been shown to prevent or minimize CIA with generally mild side effects, although the effectiveness may vary depending on myriad factors including application system, patient-level factors, and assessment tools used [11–17]. Commercially available scalp cooling systems in the USA include machine-based systems such as Paxman Scalp Cooling system [18] and the DigniCap system [19] or manual systems such as Arctic Caps [20], Chemo Cold Caps [21], and Penguin [10]. In machine-based systems, the caps are attached to a dedicated refrigeration system and one or two patients can be independently treated at a time, with cooling times of 30 min prior to infusion, during the infusion and up to 90–120 min following infusion [18, 19].

For manual cold capping systems, patients rent or buy a set of caps that are frozen in a biofreezer at the infusion center or in personal freezers using dry ice [22]. Because the caps are not connected to a cooling system, they must be changed periodically; however, patients also have increased freedom/mobility compared to machine-based systems, given that post-infusion capping lasts for up to 7 h [22]. Given the potential benefits of manual capping, we explored manual options for use at our institution and selected Penguin Cold Caps, London, UK. Penguin Caps contain a proprietary gel that maintains cold temperatures for long periods of time and do not require connection to refrigeration systems [10]. As such, patients use a set of three caps and do not have to change the caps as frequently during a session [10]. For our institutional cold capping protocol, all patients start wearing the cold cap 50 min prior to starting each chemotherapy infusion and continue to wear the cap for the entirety of each infusion. Caps are not worn between infusions. The length of time that the patient wears the cold cap after the end of each chemotherapy infusion session ranges from 3 to 7 h, depending on the chemotherapy regimen. This 3–7-h range is in contrast to machine-based capping protocols, which have post-infusion cooling times of 90–120 min. The post-infusion cooling time for manual systems may be substantially longer than machine-based systems. However, the patients continue the cold capping at home once the chemotherapy is complete, which has benefits of freeing chair space in the infusion center, keeping patients in the infusion center for less time, and allowing more patients to utilize cold capping.

Use of Penguin Caps has an overall 20–90% success rate, defined as hair loss < 50%, across several trials evaluating success in breast cancer [9, 11, 22, 23] and 79% success in a limited evaluation in cancer types [23]. The range of success rate is due, in part to measures of success (patient-reported vs. objective measures) and regimens used, with better success in non-anthracycline-based, shorter regimens [23]. It is also unclear from literature how much effectiveness varies based on patient characteristics such as hair type.

As such, the purpose of this retrospective, descriptive study was to evaluate data that are routinely recorded during the cold capping process to determine tolerability and subjective effectiveness of cold capping in minimizing CIA. We further sought to determine whether CIA varies according to chemotherapy regimens or patient/hair characteristics prior to starting chemotherapy/capping, including race, ethnicity, and hair thickness, coarseness, length, and type (e.g., straight vs. curly).

## Methods

This retrospective cohort was collected by OhioHealth and Over My Head (OMH) Boutique. OhioHealth is a large not-for-profit healthcare system that serves urban, suburban, and rural populations in Central Ohio, USA. We refer cold capping patients to OMH, which is a Medicare-certified facility located within our Cancer Center. The study was reviewed and approved by the OhioHealth Institutional Review Board (IRB1839459) with a waiver of informed consent.

The study sample included adult patients who presented to OMH to discuss cold capping between January 14, 2019 (the beginning of the cold capping program), and March 31, 2022. Exclusion criteria for the study included patients being treated for blood cancers.

Data collected included demographics, diagnoses, chemotherapy regimens, and hair characteristics (length, thickness, coarseness, type). This information is collected as part of the OMH standard processes for cold capping patient intake and monitoring during the cold capping process and was directly abstracted from OMH clinical records using a standardized data collection tool built in Research Electronic Data Capture (REDCap) [24, 25]. The primary and secondary outcomes included cold capping completion rate and the subjective percentage of hair maintained, based on patient and cold capping provider perceptions recorded during the cold capping process. To determine whether perception of hair maintained differed across patient characteristics (age, race, hair characteristics, cancer type), patients were categorized as having a favorable response (i.e., ≥ 50% hair retention) or unfavorable response (< 50% hair retention) based on previously published literature and compared using Fisher’s exact test. *P* values < 0.05 were considered statistically significant. Analyses were conducted using R Statistical Software Version 4·1·2.

## Results

Only one patient who presented to OMH to discuss cold capping during the study period decided not to pursue cold capping. The majority of patients who initiated cold capping were 31–50 years of age, Caucasian, and had a breast cancer diagnosis (Table 1).

**Table 1:** Patient Demographics and Characteristics for Patients who Initiated Cold Capping

Demographic	*N* = 101
Age, years, n (%)^a^	
18–30	17 (17·0%)
31–50	61 (61·0%)
51–70	19 (19·0%)
71–89	3 (3·0%)
Sex, n (%)	
Female	99 (98·0%)
Male	2 (2·0%)
Race, n (%)^b^	
African American	14 (14·3%)
Asian	7 (7·1%)
Caucasian	77 (78·6%)
Ethnicity, n (%)^a^	
Hispanic	6 (6·0%)
Not Hispanic	94 (94·0%)
Cancer Type, n (%)^b^	
Breast	76 (77·6%)
Gynecologic	20 (20·4%)
Prostate	1 (1·0%)
Other	1 (1·0%)

^a^Excludes 1 subject with missing data

^b^Excludes 3 subjects with missing data

The most common chemotherapy regimens for those undergoing cold capping were paclitaxel-containing (48.5%) and docetaxel-containing (35.6%) regimens, followed by doxorubicin-containing (14.9%) and cisplatin (1.0%) regimens as shown in Table 2.

**Table 2:** Chemotherapy Regimens for Patients who Initiated Cold Capping

Chemotherapy Regimen, n (%)	*N* = 101
Paclitaxel-containing^a^	49 (48.5)
Docetaxel-containing^b^	36 (35.6)
Doxorubicin-containing^c^	15 (14·9)
Cisplatin	1 (1·0)

^a^Paclitaxel; paclitaxel/trastuzumab; paclitaxel/carboplatin

^b^Docetaxel/carboplatin/trastuzumab/pertuzumab; docetaxel; docetaxel/cyclophosphamide

^c^Doxorubicin/cyclophosphamide/paclitaxel; doxorubicin/cyclophosphamide, paclitaxel/carboplatin/pembrolizumab

The hair characteristics of patients prior to starting chemotherapy are described in Table 3. There was broad distribution of hair types based on thickness, length, and type (e.g., straight, wavy, curly).

**Table 3:** Hair Characteristics Prior to Starting Chemotherapy/Cold Capping

Characteristic	*N* = 101
Hair Thickness, n (%)^a^	
Thick	33/98 (33·7%)
Thin	38/98 (38·8%)
Average	27/98 (27·6%)
Hair Coarseness, n (%)^b^	
Coarse	13/37 (35·1%)
Not Coarse	24/37 (64·9%)
Hair Type, n (%)^c^	
Straight	31/95 (32·6%)
Curly	22/95 (23·2%)
Wavy	41/95 (43·2%)
Other	1/95 (1·0%)
Hair Length, n (%)^d^	
Short	28/91 (30·8%)
Medium	39/91 (42·9%)
Long	24/91 (26·4%)

^a^Excludes 3 subjects with missing data

^b^Excludes 64 subjects with missing data

^c^Excludes 6 subjects with missing data

^b^Excludes 10 subjects with missing data

Most patients (95.0%) who initiated cold capping completed the series. Of the five patients who discontinued cold capping, the majority (80.0%) reported discomfort as a concern during the process. As such, we further explored the association between discomfort and discontinuation of cold capping. Discomfort data were available for only 19/101 subjects (Table 4). Of those 19, more patients with severe discomfort discontinued cold capping (66.7% discontinuation) than those with mild/moderate discomfort (13.3% discontinuation), although sample sizes were small, and the difference was not statistically significant.

**Table 4:** Patient-Reported Discomfort during Cold Capping and Association with Cold Capping Discontinuation, Among those with Available Data

Level of Discomfort, n (%)	*N* = 19	Cold Capping Stopped	*P*-value
Minimal/mild	15 (78·9%)	2/15 (13·3%)	0·1783
Moderate	1 (5·3%)	0 (0·0%)	
Severe	3 (15·8%)	2/3 (66·7%)	

**Fig. 1:**
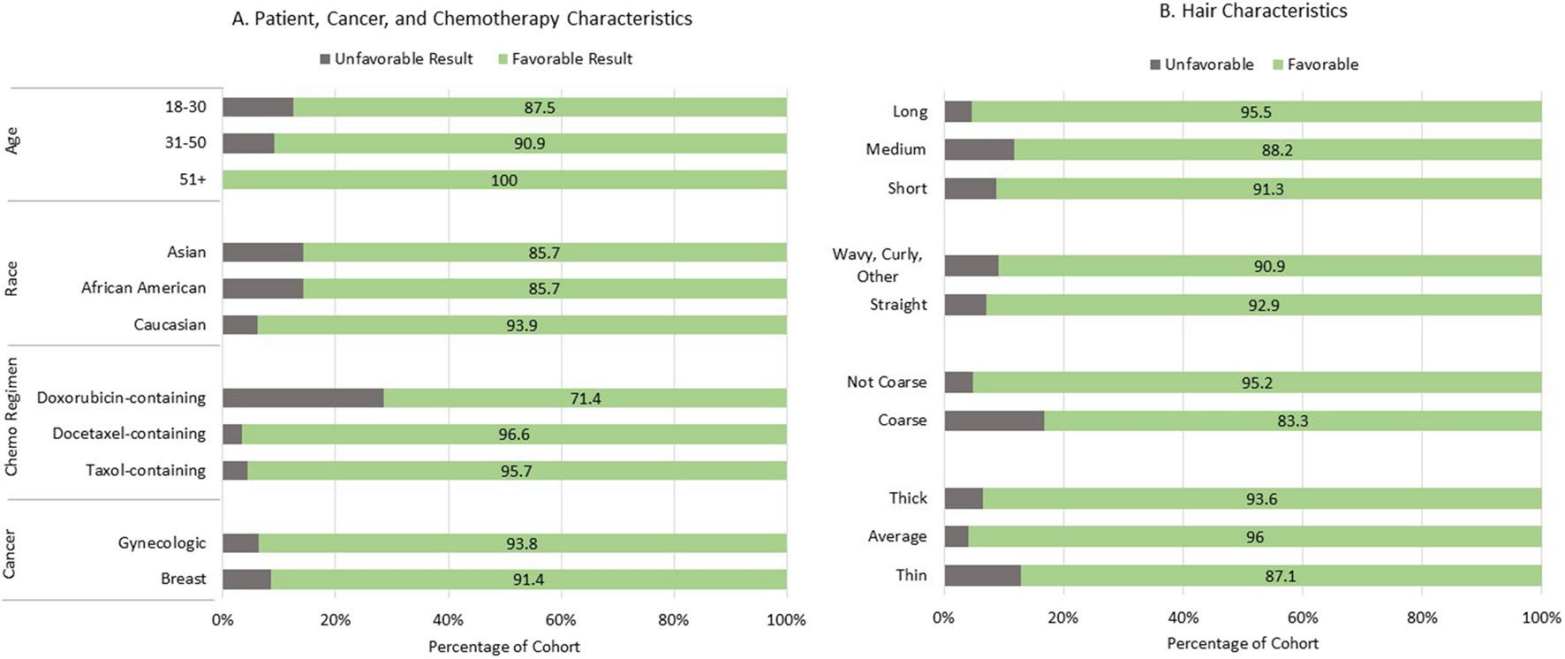
Percentage of patients with favorable vs. unfavorable cold capping results. A favorable result was defined as ≥ 50% of hair maintained. The majority of patients had a favorable result. The only factor statistically associated with favorable result was chemotherapy regimen, with favorable results noted for 71·4% of patients who received doxorubicin-containing regimens, compared to 95·7% for those who received paclitaxel-containing regimens and 96·6% for those who received docetaxel-containing regimens (*p* = 0·018). There was no apparent difference in the proportion of patients who had a favorable result based on age, race, hair thickness, coarseness, hair type, length, or cancer type

Perception of the amount of hair retained was available for 89 of the 101 cold capping patients. The median-reported percentage of hair maintained was 75%, and 82/89 (92.1% of patients) had favorable results, defined as ≥ 50% of hair retained. Of the remaining seven, percentage of hair retained was 10% (*n* = 2), 25% (*n* = 4), and 40% (*n* = 1). As shown in Fig. 1, the only factor statistically associated with favorable result (≥ 50% of hair retained) was chemotherapy regimen, with favorable results noted for 71.4% of patients who received doxorubicin-containing regimens, compared to 95.7% for those who received paclitaxel-containing regimens and 96.6% for those who received docetaxel-containing regimens (*p* = 0.018). There was no apparent difference in the proportion of patients who had a favorable result based on age (*p* = 0.5073), race (*p* = 0.2571), hair thickness (*p* = 0.6138), coarseness (*p* = 0.5381), hair type (*p* = 1.00), length (*p* = 0.8794), or cancer type (*p* = 1.00).

## Discussion

Receiving a diagnosis of cancer can be devastating to patients. A number of patients will require treatment with chemotherapy, often with a side effect of alopecia. Alopecia can affect patient’s self-esteem, body image, sexuality, and quality of life [8]. Although not a life-threatening side effect, it is a constant reminder to the patients and others that they have cancer [26]. Indeed, a small percentage of women may choose not to treat their breast cancer with chemotherapy due to the side effect of alopecia [7–9].

Cold cap therapy is an increasingly recognized non-pharmacologic intervention for reducing CIA. Historically, cold cap access has been somewhat limited as infusion centers offering scalp cooling are geographically disparate and a substantial percentage of patients live intermediate to long distances from infusion centers [27]. Like many institutions, our patients interested in cold capping had to navigate the process on their own before we implemented a formalized program. They had to research the options that were available to them, find where to get the cold capping equipment, and navigate financial impacts and other logistics of capping. OhioHealth realized that this was an opportunity to help patients maintain some control of the chemotherapy-induced alopecia and partnered with OMH to create a program offering cold capping to chemotherapy patients. We chose Penguin Cold Caps for several reasons, including portability/flexibility compared to machine-based cooling system and need for fewer caps than other manual brands [10]. Effectiveness of machine-based and manual cooling systems is highly variable. While overall it seems that more than half of all participants using scalp cooling have positive results, there are estimates as low as 8% and as high as 94% having a successful result from machine-based systems and 20 to 94% having success with manual systems [9, 11, 22, 23]. With variable literature-reported effectiveness of cold capping in reducing chemotherapy-induced alopecia, the purpose of this study was to explore the real-world effectiveness in our local setting over the first 3 years of the program, including impact of cold capping with ethnic/racial hair types.

In three years, 102 patients were referred (by physician or self) to discuss cold capping, and all but one initiated cold capping. The majority of patients had favorable results, although consistent with other literature reports of anthracycline-based regimens, patients who received doxorubicin-containing regimens had less favorable outcomes than those who received paclitaxel-containing or docetaxel-containing regimens (71.4% with favorable outcome vs. 95.7% with favorable outcome, respectively) [23, 26]. Even so, the overall effectiveness (92.1%) in this study is consistent to higher than many literature reports. One possible reason for the high success in our cohort is compliance with cold capping protocols, meaning applying the cap in the appropriate manner and wearing the cap for the prescribed durations, which may impact effectiveness [9]. This interpretation is speculative because we cannot determine full compliance due to the retrospective nature of the study. However, the partnership with OMH ensured that all patients were well-instructed in cap use, and we saw a very high completion rate (95%), with only five patients electing to stop cold capping. Providing detailed instruction on cold capping including partnership with trained cold cappers has been shown to improve patient compliance and outcomes in other studies [9, 28]. Further, this study was based on subjective reports of hair loss rather than objective measures. There are multiple reported measures for CIA [29], including Hair Mass Index [30], Visual Analog Scales, World Health Organization scale for hair loss, and Dean’s scale, as summarized in Friedrichs and Carstensen, 2014 [31]. The majority of clinical trials utilize modified Dean scale, whereby an evaluator assigns a grade between 0 (no hair loss) and 4 (> 75%) hair loss [31]. Agreement between patient-rated loss and provider, research staff, or others is variable, as is agreement between subjective reports and more objective measures. We would posit, however, that patient perceptions/satisfaction are important in real-world setting given the psychosocial effects of hair loss.

We found no significant difference in amount of hair retained based on age, race, cancer type, or hair characteristics. This information should be interpreted cautiously, as the majority of patients in our cohort were white, non-Hispanic. Of the 101 who used cold capping, only 14 reported as African American and only seven as Asian. The percentage of those with favorable response was lower for both of these patient groups (87.5% with favorable response) than for Caucasian patients (93.9%). There is extremely limited published literature on effectiveness of cold capping in other racial or ethnic populations, with those that do exist reporting results only from machine-based cooling systems. There is one report of similar results for Asian hair type vs. Caucasian [32, 33], but most of the limited reports suggest potentially less effectiveness [34, 35], with hair loss and/or alopecia-related distress actually increasing with cold capping for some groups, particularly African Americans [36, 37, 38]. Our data, while gathered from a small group of patients, may support slightly lower effectiveness in non-Caucasian patients independent of chemotherapy regimen, as none of the African American or Asian patients received doxorubicin-containing regimens.

## Limitations

There are several limitations to this study. The study sample is small, derived by convenience sampling of all available patients since the inception of the cold capping program; thus, the sample may not be representative of the general population and prone to selection bias. In particular, this study is comprised of mainly women undergoing breast cancer treatment so may not be generalizable to men or other cancer types. This study was a retrospective review of data originally collected during the course of clinical care; clinical data may be recorded differently across patients or be incomplete. There is no current standardized/systematic instrument to assess hair characteristics or alopecia for OhioHealth patients undergoing chemotherapy. This precludes our ability to include a formal control group, comparing CIA between those who did and did not undergo cold capping. Rather, data for this study were based on patient and OMH staff subjective perceptions expressed to and entered into the clinical records by cold capping staff. As such, we are limited in making direct comparisons to many literature reports of effectiveness. We do note that there needs to be more research with minorities and different hair types. There also needs to be more standard questions regarding degrees of hair loss and patient perspectives, such as use of the Chemotherapy Alopecia Distress Scale or other measures of wellness and quality of life.

## Conclusion

Chemotherapy-induced alopecia is a constant reminder to patients that they have cancer [26]. By providing a cold capping program at OhioHealth, we are empowering the individual to maintain some control of their life and be an active participant in their cancer treatment with a program that appears to have equivalent or better outcomes than other capping systems.

## Data Availability

The Data Dictionary and de-identified individual-level patient data may be provided to researchers who provide a detailed proposal for how the data will be used. Data will be available for 2 years after publication. The proposed use will be approved by the corresponding author and institution, and data will be shared following execution of an appropriate data sharing agreement. Please send enquiries to the corresponding author.

## Declarations

### Research in context

Evidence before this study

We searched EBSCOhost using the terms “cold capping OR cold cap AND penguin cold cap AND breast cancer” between January 2013 and July 2023. The search ended with 23 results. Many of the articles reviewed a manual cold capping system versus a machine-based cold capping system or using a machine-based cold capping system. The results from the studies did show favorable outcomes with using a cold capping system to reduce chemotherapy-induced alopecia.

### Added value of this study

Our institution reviewed multiple cold capping systems and ended up choosing Penguin Cold Caps. Some of the reasons are portability and flexibility than machine-based cold capping systems. The outcomes in our study were higher than the current literature.

### Implications of all the available evidence

We had a partnership with Over My Head boutique, which is one reason for the high success in our cohort. The patients had a higher compliance with cold capping protocols, meaning applying the cap in the appropriate manner and wearing the cap for the prescribed durations, which may impact effectiveness.
